# Association of serum fatty acid and estimated desaturase activity with hypertension in middle-aged and elderly Chinese population

**DOI:** 10.1038/srep23446

**Published:** 2016-03-23

**Authors:** Bo Yang, Fang Ding, Feng-Lei Wang, Jing Yan, Xiong-Wei Ye, Wei Yu, Duo Li

**Affiliations:** 1 Department of Food Science and Nutrition, Zhejiang University, Hangzhou, China; 2 The Province Center for Cardio-Cerebral-Vascular Disease, Zhejiang Hospital, Hangzhou, China

## Abstract

We aimed to investigate the cross-sectional associations of serum fatty acid (FA) and related Δ-desaturase with hypertension among 2,447 community-dwellers aged 35–79 years living in Zhejiang Province, China. Individual FA was determined in serum, Δ^5^-desaturase (D5D) and Δ^6^-desaturase (D6D) activities were indirectly estimated by FA product/precursor ratios. Participants in the highest quartile of D5D component scores (20:4n–6, 20:5n–3, 22:6n–3 and D5D) have significantly lower odds of hypertension compared with individuals in the lowest (multivariate-adjusted odds ratio (OR) = 0.68, 95% CI: 0.46–0.98). When further stratified by gender, high D5D component scores were significantly associated with lower odds of hypertension in women (OR = 0.53, 95% CI: 0.35–0.80), but not in men (OR = 0.78, 95% CI: 0.52-1.18). Multivariate-adjusted prevalent OR for an interquartile increment of individual FA and estimated desaturase was 1.27 (95% CI: 1.08–1.50) for 16:0, 1.15 (95% CI: 1.01–1.30) for 16:1n–7, 0.89 (95% CI: 0.80–0.99) for 22:6n–3, 1.32 (95% CI: 1.01–1.72) for D6D (18:3n–6/18:2n–6), and 0.74 (95% CI: 0.56, 0.98) for D5D (20:4n–6/20:3n–6). Present findings suggested that high serum 22:6n–3 and D5D as well as low 16:0, 16:1n–7 and D6D were associated with a low prevalence of hypertension in this Chinese population.

Hypertension is a major public-health challenge because of its high prevalence and concomitant cardiovascular disease (CVD) mortality and morbidity worldwide. Coinciding with food and lifestyle changes arising from industrialization and urbanization in developing countries, about 30% of adults have suffered from hypertension in China^1^. Convincing evidence from numerous meta-analyses and clinical studies suggest that increased consumption of dietary n-3 polyunsaturated fatty acid (PUFA) has been associated with decreased blood pressure (BP). It is difficult to accurately estimate the dietary intake of individual fatty acid (FA) from a FFQ or from weighed food records in most observational studies, because the database for the individual FA content of foods is not available for all foods, especially for Chinese dishes and manufactured foods, or is not up-to-date in China. FA levels in circulating blood can be regarded as a helpfully complementary tool for FFQ to closely reflect the true dietary intake of an individual. Examples of this are serum 20:5n–3 and 22:6n–3 were both significantly correlated with dietary reports of fish consumption^2^, while serum levels of 14:0, 15:0 and 17:0 were correlated with higher dairy-fat intake^3^. Circulating levels of individual FA can also be affected by endogenous desaturation catalyzed by the hepatic Δ-desaturase. Human Δ^9^-desaturase (D9D), usually referred to as stearoyl-CoA desaturase (SCD), can synthesize monosaturated FA (MUFA) from saturated FA (SFA), whereas Δ^5^-desaturase (D5D) and Δ^6^-desaturase (D6D) can catalyze the synthesis of long-chain PUFA. Because of practical and ethical reasons, hepatic desaturase activities in humans only can be indirectly estimated by FA product-to-substrate ratio from circulating lipids of fasting blood samples^4^.

Experimental studies have suggested that alterations of PUFA as components in membrane phospholipids can be partly involved in the pathogenesis of hypertension by competing for eicosanoid metabolic pathway to change the balance of vasodilator/vasoconstrictor, the systemic arterial compliance mediated by the release of nitric oxide (NO)^5^ and the voltage dependent L-type Ca^2+^ channel levels^6^. Many observational studies are rather consistent in demonstrating that high circulating 16:0, 16:1n–7, 20:3n–6 and low 18:2n–6 are independently related to cardiometabolic diseases^7^,^8^,^9^. Nevertheless, to date, no study systematically evaluated the role of Δ-desaturase in the development of clinical hypertension, and the association among Chinese is presently unclear. Thus, we analyze baseline data from 2447 individuals (1153 males and 1294 females) aged 35–79 years who participated a community-based cohort in Zhejiang Province, China to investigate the cross-sectional associations of serum fatty acid (FA) and related Δ-desaturase with prevalent hypertension in this middle-aged and elderly Chinese population.

## Results

### Baseline characteristics

The demographic and clinical characteristics of participants were showed in Supplementary Table S1. The 2447 participants (mean age of 55.13 y) included 1153 males (47.12%) and 1294 females (52.88%), 802 overweight individuals (32.77%), 839 obese individuals (34.29%), 601 smokers (24.19%), 666 drinkers (27.22%), 136 highly educated individuals (5.25%), 915 manual laborers (38.66%), 588 regular exercisers (23.85%), 494 participants with high consumption of salt (19.50%), 891 persons with a dietary history of animal oil (36.41%), 597 individuals with a family history of hypertension (24.39%), 847 individuals with high HR levels (34.61), 734 individuals with high TG (30.00%), 668 individuals with high TC (27.30%), and 79 individuals with diabetes mellitus (DM). Baseline characteristic for 2447 individuals by tertiles of SFA, MUFA and PUFA was presented in Table 1, respectively. The proportions of males significantly increased with serum compositions of SFA (*P* for trend = 0.020) and MUFA increasing (*P* for trend = 0.030), but significantly reduced with PUFA increasing (*P* for trend = 0.011). Participants in the highest tertiles of serum SFA and MUFA were more likely to be younger, obese, current smokers, manual workers, high BP, high TC, high TG and DM, whereas participants in the highest tertiles of serum PUFA were more likely to be elder, highly educated and regular exercisers when compared with individuals in the lowest tertiles.

**Figure 1:**
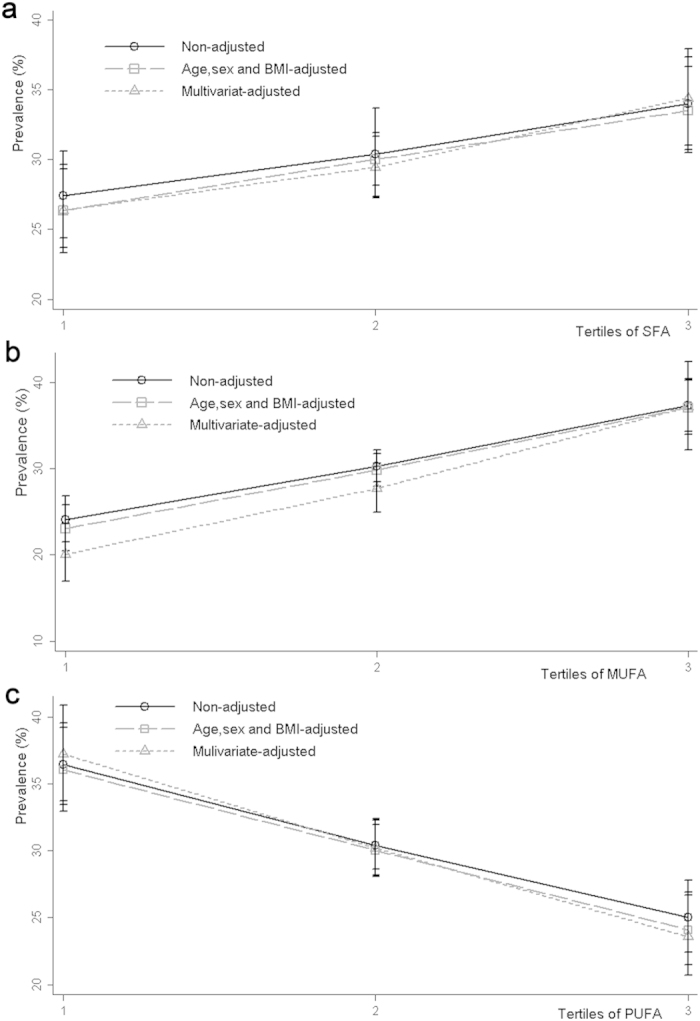
The prevalence of hypertension by tertiles of serum SFA, MUFA and MUFA in the middle-aged and elderly Chinese population. The prevalence of hypertension by tertiles of serum compositions of SFA, MUFA and MUFA was shown in figure (a–c), respectively. The prevalence of hypertension with 95% confidence interval (CI) and corresponding *P* for trend across increasing tertiles were estimated by non-adjusted logistic regression model, age, gender and BMI-adjusted model (all *P* for trend < 0.01) and multivariate-adjusted model (all *P* for trend < 0.01), respectively. The prevalence across each tertile was separately represented by black circles, squares and triangles, and error bars denoted corresponding 95% CI.

The prevalence of hypertension was 30.57% in 2447 participants, with no significant difference by gender (χ^2^ = 0.440, *P* = 0.507). The prevalence was 33.42% in middle-aged and 27.93% in elderly participants, with a significant difference by age (χ^2^ = 8.54, *P* = 0.010). In unadjusted analyses, the prevalence of hypertension significantly increased across increasing tertiles of SFA and MUFA, but decreased across increasing tertiles of PUFA (all *P* for trend < 0.01). The trend association remained significant in age, BMI and gender-adjusted models and multivariate-adjusted models (Fig. 1).

### FA compositions and related desaturase activities between participants with and without hypertension

The total 748 hypertension patients (360 men and 388 women) were identified from 2447 individuals with fasting blood samples, with 308 cases identified by elevated SBP or DBP only and the remaining cases identified by self-reported treatment of HT with anti-hypertensive drugs only. The difference in serum FA and estimated desaturase pattern between hypertensive and normotensive participants was shown in Table 2. Hypertensive participants had significantly higher serum 16:0, 16:1n–7, 18:1n–9, 20:3n–6, SCD-1, SCD-2 and D6D as well as lower 22:6n–3, 18:2n–6 and D5D compared with normotensive participants. Even after controlling for age, BMI and gender, the difference in serum FA profile remain significant between hypertensive and normotensive participants.

The difference in FA compositions and estimated desaturase pattern between hypertensive and normotensive participants stratified by gender was presented in Supplementary Table S2. Women patients with hypertension had significantly higher serum 16:0, 18:0, 18:3n–6, 20:3n–6 and D6D activity as well as lower 18:3n–3 compared with normotensive women, but the difference in serum FA and related desaturase pattern was not found in hypertensive men compared with normotensive men.

### Principal component loading of individual FA and estimated desaturase index

**Figure 2:**
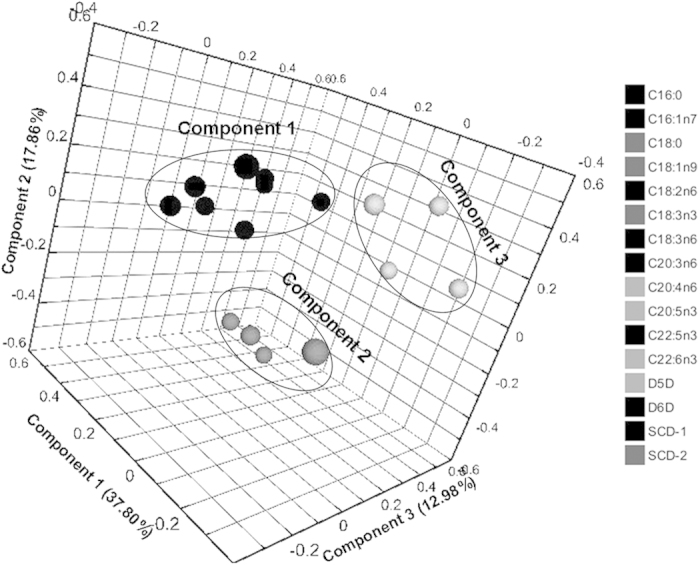
Rotated loading plot of individual FA and estimated desaturase index by principal component analyses. Varimax rotation loading of individual FA and estimated desaturase index revealed 3 major FA-components: Comp 1, Comp 2 and Comp 3. Comp 1 (D6D component) comprised higher positive loadings from serum 16:0, 16:1n–7, 18:3n–6, 20:3n–6, 22:5n–3, SCD-1 and D6D as well as a negative loading from 18:2n–6. Comp 2 (SCD-2 component) mainly comprised higher positive loadings from SCD-2 and 18:1n–9 as well as negative loadings from 18:3n–3 and 18:0. Comp 3 (D5D component) mainly comprised higher loadings from serum 20:5n–3, 20:4n–6, 22:6n–3 and D5D.

The three significantly principal FA-components that explained 68.64% of individual FA-related variance were shown in Fig. 2. Rotated varimax loading of individual FA and desaturase index can be found as Supplemental Table S3. Comp 1 (eigenvalue = 3.42) comprised higher positive loadings from 16:0, 16:1n–7, 18:3n–6, 20:3n–6, 22:5n–3, SCD-16 and D6D as well as a negative loading from 18:2n–6, which can be defined as “D6D component”. Comp 2 was defined as “SCD-2 component” (eigenvalue = 2.78), which mainly comprised higher positive loadings from SCD-2, 18:1n–9 and 18:3n–3 as well as a negative loading from 18:0. Comp 3 was defined as “D5D component” (eigenvalue = 1.46), which mainly comprised higher positive loadings from 20:5n–3, 20:4n–6, 22:6n–3 and D5D.

### Principal FA-component and prevalent hypertension

Associations between the major FA-components and prevalent hypertension were presented in Table 3. When comparing the highest quartile (Q_4_) of FA-component scores with the lowest quartile (Q_1_), D6D component (Comp 1) was positively associated with prevalent hypertension (OR = 1.78, 95% CI: 1.39–2.28, *P* for trend < 0.001). The association remained significant in model 1 adjusted for age, BMI and gender, model 2 additionally adjusted for lifestyle factors and model 3 adjusted for covariates in model plus clinical factors, respectively. SCD-2 component (Comp 2) was positively associated with prevalent hypertension in crude model, model 1 and model 2, respectively. After additional adjustment for clinical factors in model 3, the association was attenuated to null. By contrast, a significantly inverse association was found between D5D component (Comp 3) and prevalent hypertension in crude model (Q_4_ vs. Q_1_: OR = 0.56, 95% CI: 0.43–0.71, *P* for trend = 0.001). The association was attenuated but remained statistically significant in model 1, model 2 and model 3, respectively (all *P* for trend < 0.05).

Multivariate-adjusted OR for prevalent hypertension in the highest compared with the lowest quartile of principal component scores stratified by gender, age and BMI was presented in Supplementary Table S4. After multiple adjustments, D6D component was positively associated with prevalent hypertension in women (OR = 1.71, 95% CI: 1.13–2.57), but not in men (OR = 1.21, 95% CI: 0.79–1.85). However, results from interaction tests showed that no significant difference was found between women and men. Similarly, D5D component was negatively associated with prevalent hypertension in women (OR = 0.53, 95% CI: 0.35–0.80), but not in men (OR = 0.78, 95% CI: 0.52–1.18), with no significant difference by gender (*P* for interaction = 0.59). When further stratified by age and BMI, these observed associations were consistent for the subgroup by age (middle-aged vs. elderly) and BMI (normal vs. overweight), and no significant interactions was found between prevalent hypertension and the strata analyzed.

### Individual FA and the prevalence of hypertension

**Figure 3:**
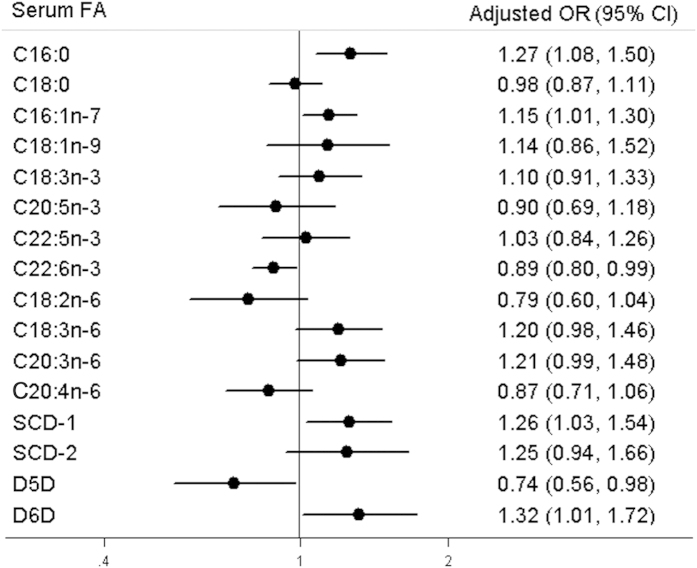
Associations of individual fatty acid and estimated desaturase index with prevalent hypertension in the middle-aged and elderly Chinese population. The multivariate-adjusted odds ratio (OR) with 95% confidence interval (CI) for prevalent hypertension in relation to an interquartile increment of individual FA and estimated desaturase index were estimated by logistic regression models adjusted for age, gender, BMI, lifestyle and clinical factors. Prevalent OR was represented by black circles, and error bars denoted corresponding 95% CI.

The multivariate-adjusted OR with 95% CI for hypertension in relation to an interquartile increment of individual FA was presented in Fig. 3. After adjustment for age, gender, BMI, lifestyle and clinical factors, OR with 95% CI for association of prevent hypertension with an interquartile increment of individual serum FA and estimated desaturase index was 1.27 (95% CI: 1.08–1.50) for 16:0, 1.15 (95% CI: 1.01–1.30) for 16:1n–7, 0.89 (95% CI: 0.80–0.99) for 22:6n–3, 1.21 (95% CI: 0.99–1.48) for 20:3n–6, 1.26 (95% CI: 1.03–1.54) for SCD–1, 0.74 (95% CI: 0.56–0.98) for D5D and 1.32 (95% CI: 1.01–1.72) for D6D, respectively.

## Discussion

To our knowledge, our study firstly evaluated the role of estimated desaturase activity in the development of hypertension, and important findings were disclosed. Serum FA and estimated desaturase pattern in hypertensive participants is typically characterized by higher 16:0, 16:1n–7, 20:3n–6, D6D and SCD as well as lower 18:2n–6, 22:6n–3 and D5D compared with normotensive participants. When further stratified by gender, higher compositions of serum 16:0, 18:0, 18:3n–6, 20:3n–6 and D6D activity as well as lower 18:3n–3 were found in hypertensive women compared with normotensive women, but not in hypertensive men compared with normotensive men. In addition, the interrelations between individual serum FA and estimated desaturase activity may partially be explained by the 3 significant FA-components (D6D, SCD-2 and D5D component) generated from PCA. In multivariable analyses of the highest quartile of FA-component scores compared with the lowest, D6D component was adversely associated with hypertension, whereas D5D component was beneficially associated with hypertension, with no significant interactions between prevalent hypertension and the strata factors by age (middle-aged vs. elderly), gender and BMI (normal vs. overweight). Increased serum 22:6n–3 and D5D as well as low 16:0, 16:1n–7, 20:3n–6 and D6D were associated with a decreased prevalence of hypertension. These observed associations cannot be affected by possible confounding factors caused by age, gender, BMI, lifestyle characteristics (smoking, drinking, salt intake, animal oil intake, profession, education, and exercise habit) and clinical factors (family history of hypertension, heart rate, blood lipid and fasting glucose levels). These data suggested that the abnormal serum FA and estimated desaturase pattern probably accompanies the development of hypertension in this middle-aged and elderly Chinese population.

The current study described the difference in serum FA profile between hypertensive and normotensive individuals, which was relatively consistent with the results from a previous study^10^. In addition, we observed significantly higher SCD and D6D as well as lower D5D in hypertensive patients than in normotensive individuals. This abnormal desaturase pattern was also found in myocardial infarction^11^, stroke^12^,^13^ and left ventricular hypertrophy^14^. The desaturase activity estimated by serum FA product-to-substrate ratio may be affected not only by endogenously metabolic factors^15^, but also by dietary fat quality^16^. The 3 major FA-components were generated from PCA model of individual FA and estimated desaturase index, which may partially be interpreted as an integrate consequence of FA intake and endogenous metabolism. These FA-components could represent a range of FA metabolic pathways rather than just the enzyme activities as such. It seemed to be surprising that D6D component as the most powerful FA-component also comprised 16:0 and 16:1n-7, but this probably indicated endogenous synthesis more so than dietary intakes. Increased proportion of 16:1n–7 in serum may be a consequence of endogenous desaturation driven by SCD-1, because of lower 16:1n–7 in diet. A possible explanation is that a diet rich in SFA will accelerate an endogenous synthesis of 16:1n–7 from 16:0 through counteracting the inhibitory effects of a PUFA-rich diet on the expression of SCD^17^.

It is known that physical exercise, smoking, and alcohol intake can change the desaturase activity^18^, yet the observed associations for the 3 major FA-components were independent of these factors. After additional adjustment for blood lipid and fasting glucose level, the associations were attenuated but remained significant only for D6D and D5D component, indicating that the association for SCD-2 component may partially be mediated by obesity-related metabolic disorders. Abnormal glucose metabolism and dyslipidemia were associated with incident hypertension, and insulin resistance may precede hypertension^19^,^20^. Animal experiments revealed that the hepatic gene expression of SCD-1 is positively correlated with insulin sensitivity in obese rats^21^,^22^. A possible explanation was that up-regulation of hepatic SCD activity increased insulin signaling via reduced phosphorylation of insulin-receptor substrates^15^, leading to stimulation of the sympathetic nervous system and inducing hypertrophy of vascular smooth muscle^23^. Furthermore, the positive loading of 18:1n–9 and SCD-2 were higher in SCD-2 component, but no significant association was observed between 18:1n–9 as a determinant subtype of serum MUFA and prevalent hypertension in the multivariable model. thus, the null observations for SCD-2 component may also be explained by a dilute endogenous synthesis caused by higher dietary consumption of 18:1n–9^24^. By contrast, the favorable results for D5D component may be attributable to the main contribution made by 20:5n–3 and 22:6n-3. There were higher positive loading of 20:5n–3, 22:6n–3 and D5D in this FA-component, which suggested that a more effective synthesis of long-chain n-3 FA from 18:3n–3 in the D5D/D6D pathway^16^. 20:4n–6 can form the precursor of 2 series of PGs that stimulates vascular constriction and smooth muscle contraction, whereas 20:5n–3 is precursor of PGI_3_ and thromboxane A_3_ that have been reported to be less active. It is possible that increased consumption of marine food rich in 20:5n–3 and 22:6n–3 can competitively inhibit hepatic D5D activity to reduce circulating levels of metabolites from 20:4n–6 such as PGF_2a_ and thromboxane A_2_.

Some strength should be highlighted in our study. Firstly, PCA was performed to capture the inner relationships between serum FA and related desaturase, facilitating the interpretation of the findings. Furthermore, the large sample size, available data on lifestyle and clinical factors, the wide range of age and the robustness in our findings are strengths of our study. Nevertheless, several limitations should also be taken into account in the present study. Firstly, baseline data analyses within a community-based cohort limit the ability to infer the causal relationships. Secondly, caution is need for generalizing the present endings to the whole Chinese population, because the community-dwellers aged 35–79 years living in Zhejiang province did not perfectly represent a random sample of the Chinese adults. Thirdly, prevalent hypertension was defined by self-reported information, possibly leading to a misclassification. However, the accuracy of self-reported hypertension has been demonstrated in previous validation studies by using clinical measurements^25^ and medical record review^26^. Fourthly, considering that data regarding FA consumption were not available for our study, it is difficult to find firm evidence that the differences in the serum FA profile between hypertensive and normotensive participants were mainly attributed to the endogenous FA synthesis driven by Δ-desaturase. Fifthly, despite comprehensive adjustment for demographic lifestyle and clinical factors in statistical models, the possibility of unmeasured or unknown confounders could not be rule out. Sixthly, although the use of a FA product to precursor ratio as a surrogate measure to estimate desaturase activity is well established in human studies, estimated D5D (20:4n–6/20:3n–6) for the n–6 PUFA pathway may not be estimating the index (20:5n–3/20:4n–3) for the n–3 PUFA pathway. Thus, conclusions about D5D activity from the n–3 PUFA pathway cannot be made. However, in contrast to the conversion of n–6 FA, the amount of n–3 FA conversion is relatively lower^27^, estimated desaturase index for n–6 PUFA passway may be useful for understanding FA desaturation pattern in hypertension. Finally, estimated desaturase index in serum triglycerides and phospholipids was closely related to those in liver. Thus, the desaturase activity estimated by FA ratios derived from total serum lipids should be interpreted with caution.

In conclusion, the serum FA and related desaturase pattern, as evaluated by principal components from PCA, was associated with prevalent hypertension. High proportion of serum 22:6n–3 and the activity of D5D as well as low 16:0, 16:1n–7 and D6D were associated with a low prevalence of hypertension in this middle-aged and elderly Chinese population. Such findings contribute to knowledge regarding the preventive and therapeutic effects of n-3 PUFA on clinical hypertension. Whether n-3 PUFA supplementation is beneficial for prevention and treatment of hypertension among Chinese needs to be determined in long-term clinical trials.

## Methods

### Study design

A cross-sectional study with baseline data was performed from a community-based cohort study in Zhejiang Province, China (Zhejiang Prospective Investigation into Hypertension and Lifestyle). All subjects were entered in the community-based cohort by a cluster random sampling in 6 neighborhoods across three cities (Hangzhou, Jiaxin and Shaoxin) located in Zhejiang Province, China. Baseline assessment included the collection of blood samples, anthropometric measurements and a face-to-face interview using a validated questionnaire about socio-demographic and lifestyle characteristics between April and October 2012.

### Participants

The study protocol was approved by the Ethics Committee, College of Biosystem Engineering and Food Science in Zhejiang University. The methods were carried out in accordance with the approved guidelines. All the subjects provided their written consent before data collection. The total 3500 community-dwelling women and men aged 35–79 years free-living in Zhejiang Province were invited to participate between April and October 2012. To define our study population, subjects were excluded if they meet any of the following items: myocardial infarction, coronary heart disease, peripheral vascular disease, angina, cancer, dementia, schizophrenia, deaf, <35 years, less than 6 months of living in the local. After the exclusions, 3200 subjects aged ≥35 years were entered in the community-based cohort. A total of 3,017 participants agreed to participate and provided written informed consent. Of those, 2,535 participants provided overnight fasting blood samples and completed a laboratory assessment, with the participation rate of 84.02% (2,535/3,017). Subjects with missing values (n = 56) or implausible data (n = 19) on lifestyle factors from the self-administered questionnaire and with unreliable data on serum FA proportions (n = 13) were excluded. Thus, 2447 participants (1153 males and 1294 females) were finally remained for baseline analyses.

### Clinical and biochemical measurements

A standard questionnaire was administered by trained investigators to collect information on socio-demographic characteristics, lifestyle, family history of hypertension and medical history. Anthropometrical measurements were performed by trained nurses using standard protocols included height (m), weight (kg), heart rate (HR, beat/min), blood pressure (BP, mm Hg). Body mass index (BMI) was calculated as the participant’s weight (kg) divided by the square of standing height (m^2^). Before BP measurement, subjects were advised to avoid consuming alcohol or tobacco, ingesting tea or coffee, and engaging in exercise for at least 30 minutes. A standardized mercury sphygmomanometer was used to measure BP by trained nurses, and one of three cuff sizes (regular adult, large or thigh) was chosen based on the circumference of the participant’s arm. BP for each subject was defined as the average of three measurements performed in the subject in sitting position with 2-min intervals at that visit. Prevalent hypertension was ascertained by meeting 1 of 3 criteria: (a) a new physician diagnosis of HT (an average systolic BP (SBP) ≥140 mm Hg and/or an average diastolic BP (DBP) ≥90 mmHg); (b) newly initiated antihypertensive treatment (hypertension duration ≤6 months); (c) self-reported treatment of hypertension with anti-hypertensive medication (hypertension duration >6 months)^28^. Fasting blood samples were collected in tubes with serum separator gel. They were left at room temperature for 30 min and then centrifuged at 2500 RCF (g) for 10 to 15 min to isolate serum. Measurement of serum triglycerides (TG), total cholesterol (TC) and fasting glucose (Fbg) levels were determined by standard procedures at the biochemistry laboratory of Zhejiang Hospital. The remaining serum samples were aliquoted into separate tubes (1 mL) and stored at −80 °C until further FA analyses.

### Fatty acid and related desaturase activities analyses

FA composition in serum was analyzed between September 2013 and June 2014.

The total lipid content of the serum was extracted with solvents^29^,^30^. The methyl esters of the fatty acids in serum were prepared by saponification, and the serum compositions of FA were determined by gas-liquid chromatography (GLC) as described previously^30^. FA compositions were expressed as a percentage of total FAs in serum. Desaturase activity was estimated as the product-to-precursor ratio of FA composition in serum according to the following: SCD-1 = 16:1n−7/16:0, SCD-2 = 18:1n–9/18:0, D5D = 20:4n–6/20:3n–6, and D6D = 18:3n–6/18:2n–6.

### Statistical analysis

Data analyses were performed by STATA version 11.0 (Stata CORP, College Station, TX). The normally distributed data were expressed as the mean ± SD (standard deviation), while the skewed data were expressed as the median ± QR (quartile range) and were log-transformed before statistic analyses. Serum FA compositions were divided into tertiles, and distribution of lifestyle and clinical factors was compared across tertiles of SFA, MUFA and PUFA, respectively. *P* for trend across tertiles of serum FA in the continuous and categorical variables was calculated by a generalized liner model (GLM) and the chi-square test, respectively. Difference in individual FA and estimated desaturase index between hypertensive and normotensive individuals was tested by a GLM.

Individual FA and estimated desaturase index were entered in principal component model (PCA) followed by variance rotation to determine the interrelation between individual FAs in serum. Component loadings of ≥|0.40| were considered meaningful for the interpretation of principal component pattern^31^. Firstly, we used three multiple logistic regression models to estimate adjusted odds ratio (OR) with 95% confidence interval (CI) for prevalent HT across quartiles of principal component scores, with the lowest quartile as the reference. Then, *P* for trend was calculated across quartiles by entering the ordinal numbers within quartiles as a continuous variable into the corresponding models. Model 1 was adjusted for age, gender and BMI, while model 2 was further adjusted for lifestyle factors including smoking, drinking, education, profession, exercise habit, slat intake and animal fat intake. Model 3as a full model was adjusted for age, gender, BMI, lifestyle factors, and clinical factors including family history of HT, anti-hypertensive drug use, heart rate, blood lipid and fasting glucose levels. Explanatory variables were modeled as following: age (35–55, 55–79), BMI (<24, 24–28, ≥28), smoking (never, former, occasional (<18 pack/year) and current (≥18 pack/year)), drinking (never, former, occasional (<25 g/day) and current (≥25 g/day)), education duration (primary ≤6 years, secondary 6–12 years, high ≥12 years), profession (manual, non-manual), exercise habit (≥3 times/week, <3times/week), salt intake (high-salt (>10 g/day), low-salt (<10 g/day)), animal oil intake (yes (≥2 times/week), no (<2 times/week)), family history of HT (yes, no), anti-hypertensive drug use (yes, no), HR (<67 beat/min, 67–74 beat/min, ≥74 beat/min; tertiles), TC (≤5.18 mmol/L, >5.18 mmol/L), TG (≤1.70 mmol/L, >1.70 mmol/L), and Fbg (<6.1 mmol/L, 6.1–7.0 mmol/L, ≥7.0 mmol/L).

Secondly, we performed analyses stratified by age (<55 y vs. ≥55 y), gender and BMI (<24 vs. ≥24) to estimate the consistency of the findings. Interaction tests were also used to determine whether prevalent OR differed between the strata analyzed by including simultaneously each strata factor, the quartiles of principal component scores and the respective interaction terms (strata factor multiplied by quartiles of principal component scores) in the models. Finally, to test robustness of the findings from PCA, the logistic regression model was repeated to estimate multivariate-adjusted OR with 95% CI for an interquartile increment of individual FA. Two-sided *P* < 0.05 was considered statistically significant.

## Additional Information

**How to cite this article**: Yang, B. *et al.* Association of serum fatty acid and estimated desaturase activity with hypertension in middle-aged and elderly Chinese population. *Sci. Rep.* **6**, 23446; doi: 10.1038/srep23446 (2016).

## Supplementary Material

Supplementary Information

## Figures and Tables

**Table 1:** Baseline characteristics of 2447 middle-aged and elderly participants by tertile (T) of serum fatty acid.

Characteristics*	T1 (<29.28)	SFA			MUFA			PUFA	
		T3 (≥31.46)	*P*, Trend#	T1 (<19.96)	T3 (≥23.79)	*P*, Trend#	T1 (<40.14)	T3 (≥44.94)	*P*, Trend#
N	815	814		812	817		814	811	
Age									
Middle-aged, %	31.95	34.58		30.48	37.26		37.51	29.97	0.55
Elderly, %	34.97	31.70	0.207	35.98	29.03	0.02	28.41	35.92	<0.01
Continues, y	56.11 ± 11.22	55.71 ± 11.42	0.47	56.79 ± 11.40	54.62 ± 11.06	< 0.01	54.41 ± 11.04	56.70 ± 11.39	0.01
Male, %	55.35	48.7	0.02	55.54	50.93	0.03	50.25	57.84	<0.01
BMI									
Overweight, %	33.79	32.29	0.31	34.38	30.38	0.28	31.09	34.46	0.08
Obesity, %	28.97	37.50 |	<0.01	28.67	37.40	<0.01	37.35	27.80	<0.01
Continues, kg/m^2^	23.52 ± 3.24	24.24 ± 3.46	<0.01	23.37 ± 3.00	24.24 ± 3.58	<0.01	24.20 ± 3.58	23.76 ± 2.95	<0.01
Lifestyle factors (%)									
Current smoking	22.37	26.11	0.02	21.29	26.02	0.05	26.99	20.05	<0.01
Current drinking	22.01	32.13	<0.01	29.03	26.82	0.11	26.77	25.09	0.31
High education	4.04	2.89	0.02	8.73	1.12 |	<0.01	0.75	7.56	<0.01
Manual labor	35.14	42.42	0.04	39.66	38.45	0.13	39.44	37.43	<0.01
Regular exercise	29.22	19.02	<0.01	29.71	16.45 |	<0.01	13.9	30.86	<0.01
High salt intake	32.14	33.40	0.81	31.57	36.03	0.26	37.21	31.08	0.10
Animal oil intake	28.97	37.32	<0.01	27.06	35.63	<0.01	35.17	28.76	0.01
Clinical factors									
Family history of hypertension, %	27.9	18.79	<0.01	18.5	28.87	<0.01	27.61	20.95	0.03
Anti-hypertensive drugs use, %	32.98	33.75	0.44	33.75	30.41	0.20	41.47	26.76	<0.01
SBP, mm Hg	124.84 ± 16.43	129.52 ± 18.78	<0.01	123.93 ± 17.63	129.08 ± 17.46	<0.01	129.25 ± 18.20	123.68 ± 17.17	<0.01
DBP, mm Hg	81.13 ± 9.34	82.03 ± 9.53	<0.01	79.90 ± 9.26	82.11 ± 9.82	<0.01	81.99 ± 9.33	80.00 ± 9.26	<0.01
HR, beat/min	71.63 ± 10.20	70.63 ± 10.61	0.08	69.58 ± 9.48	72.80 ± 0.33	<0.01	72.39 ± 11.00	69.97 ± 9.56	<0.01
TG									
High TG, %	26.51	42.92	<0.01	21.23	49.85	<0.01	47.83	22.79	<0.01
Continuous, mmol/L	1.48 ± 0.18	1.74 ± 0.26	<0.01	1.28 ± 0.15	1.95 ± 0.31	<0.01	1.95 ± 0.41	1.34 ± 0.14	<0.01
TC									
High TC, %	29.30	33.94	0.060	36.92	29.47	0.48	27.56	39.60	0.01
Continuous, mmol/L	4.66 ± 0.96	4.74 ± 1.06	0.22	4.59 ± 0.95	4.68 ± 0.92	0.04	4.59 ± 0.96	4.83 ± 1.06	<0.01
Fbg									
IFG, %	25.45	45.45	0.06	37.04	33.33	0.83	35.19	37.04	0.65
DM, %	20.83	41.67	0.04	36.11	30.56	0.98	34.72	29.17	0.60
Continuous, mmol/L	4.72 ± 1.16	4.82 ± 0.94	<0.01	4.88 ± 1.09	4.77 ± 1.23	0.06	4.80 ± 1.21	4.87 ± 1.00	0.24

N, number of subjects; HR, heart rate; TG, triglyceride; TC, total cholesterol; IFG, impaired fasting glucose; DM, diabetes mellitus.

^*^Continuous data were expressed as the mean value ± SD (standard deviation), while categorical data were expressed as proportions (%).

^#^*P* for trend across tertiles of serum FA in the continuous and categorical variables was examined by a generalized linear model and the chi-square test, respectively.

**Table 2:** Serum fatty acid and estimated desaturase index in 2447 individuals by hypertension status.

Fatty acid and desaturase index*	Normotensive (n = 1699)	Hypertensive (n = 748)	*P* value#	*P* value†
16:0	20.62 ± 2.60	21.05 ± 2.57	<0.01	<0.01
18:0	6.40 + 0.85	6.34 ± 0.78	0.04	0.08
16:1n–7	1.52 + 0.78	1.69 ± 0.82	<0.01	<0.01
18:1n–9	18.95 ± 3.75	19.98 ± 3.75	<0.01	<0.01
18:3n–3	0.93 ± 0.41	0.94 ± 0.37	0.06	0.10
20:5n–3	3.35 ± 1.60	3.31 ± 1.55	0.44	0.36
22:5n–3	0.46 (0.38–0.56)	0.47 (0.38–0.58)	0.53	0.42
22:6n–3	1.74 ± 0.69	1.65 ± 0.51	<0.01	0.01
18:2n–6	28.52 ± 5.28	27.40 ± 5.21	<0.01	<0.01
18:3n–6	0.32 (0.20–0.46)	0.36 (0.25–0.51)	<0.01	0.01
20:3n–6	1.13 ± 0.31	1.16 ± 0.32	0.02	0.04
20:4n–6	6.01 ± 1.62	6.04 ± 1.56	0.12	0.27
SCD-1 (×10^−2^)	6.60 (4.91–8.92)	7.35 (5.41–9,98)	<0.01	<0.01
SCD-2	3.00 ± 0.75	3.18 ± 0.76	<0.01	<0.01
D5D	5.66 ± 0.96	5.20 ± 0.87	<0.01	0.01
D6D (×10^−2^)	1.13 (0.73–1.77)	1.34 (0.84–2.05)	<0.01	0.01

SCD-1, 16:1n–7/16:0; SCD-2, 18:1n–9/18:0; D5D, 20:4n–6/20:3n–6; D6D, 18:3n–6/18:2n–6.

^*^Data with normal distribution were expressed as the mean (s.d.), while the skewed data were expressed as the median (quartile rage).

^#^*P* value between groups was calculated by a non-adjusted GLM.

^†^*P* value between groups was calculated by age, BMI and gender-adjusted GLM.

**Table 3:** Associations between the major FA-components and prevalent hypertension in 2447 middle-aged and elderly participants.

Component	case/n	Crude Model*		Model 1#		Model 2†		Model 3§	
		OR (95% CI)	*P* value	OR (95% CI)	*P*value	OR (95% CI)	*P*value	OR (95% CI)	*P* value
Comp 1 (D6D component)									
Q_1_	150/611	1.00 (Ref.)		1.00 (Ref.)		1.00 (Ref.)		1.00 (Ref.)	
Q_2_	179/612	1.27 (0.98–1.63)	0.064	1.17 (0.90–1.52)	0.2300	0.94 (0.64–1.38)	0.7300	1.00 (0.69–1.48)	0.8590
Q_3_	194/611	1.43 (1.11–1.84)	0.005	1.33 (1.03–1.72)	0.0270	1.10 (0.74–1.63)	0.5900	1.12 (0.76–1.64)	0.5680
Q_4_	225/613	1.78 (1.39–2.28)	<0.001	1.70 (1.32–2.18)	0.0000	1.46 (1.02–2.12)	0.0430	1.42 (1.00–2.09)	0.0490
*P* for trend		0.000		0.000		0.025		0.039	
Comp 2 (SCD-2 component)									
Q_1_	147/611	1.00 (Ref.)		1.00 (Ref.)		1.00 (Ref.)		1.00 (Ref.)	
Q_2_	183/613	1.34 (1.04–1.73)	0.023	1.28 (0.99–1.67)	0.0560	1.26 (0.90–1.76)	0.2300	1.23 (0.87–1.74)	0.2400
Q_3_	195/613	1.47 (1.14–1.89)	0.003	1.36 (1.05–1.76)	0.0200	1.27 (0.89–1.70	0.0270	1.17 (0.81–1.69)	0.4010
Q_4_	223/610	1.82 (1.42–2.33)	<0.001	1.69 (1.31–2.18)	0.0000	1.51 (1.02–1.20)	0.0000	1.42 (0.93–2.17)	0.1070
*P* for trend		0.000		0.001		0.036		0.137	
Comp 3 (D5D component)									
Q_1_	208/611	1.00 (Ref.)		1.00 (Ref.)		1.00 (Ref.)		1.00 (Ref.)	
Q_2_	184/613	0.72 (0.56–0.92)	0.008	0.73 (0.57–0.94)	0.0140	0.81 (0.58–1.45)	0.2400	0.87 (0.62–1.24)	0.3350
Q_3_	193/612	0.70 (0.54–0.90)	0.007	0.75 (0.59–0.96)	0.0240	0.77 (0.54–1.08)	0.1200	0.81 (0.56–1.17)	0.2100
Q_4_	163/611	0.56 (0.43–0.71)	0.001	0.60 (0.46–0.78)	0.0010	0.65 (0.46–0.96)	0.0330	0.68 (0.46–0.98)	0.0240
*P* for trend		0.001		0.014		0.030		0.041	

OR, odds ratio; CI, confidence interval; Ref. reference; Q, quartile.

^*^Non–adjusted model.

^#^Adjusted for age, BMI and gender.

^†^Additionally adjusted for lifestyle factors (smoking, drinking, education, profession, physical exercise, salt intake and animal oil intake).

^§^Additionally adjusted for clinical factors (family history of hypertension, TC, TG, Fbg and heart rate).
