# Supplementary material for: Association of serum fatty acid and estimated desaturase activity with hypertension in middle-aged and elderly Chinese population
Source: Sci Rep. 2016 Mar 23;6:23446. doi: 10.1038/srep23446 (PMC4804219; doi:10.1038/srep23446)
Supplement: Supplementary Information [file srep23446-s1.pdf]

Association of serum fatty acid and estimated desaturase activity with hypertension in middle-aged and elderly Chinese population

Bo Yang[1+], Fang Ding[2+], Feng-Lei Wang[1], Jing Yan[2], Xiong-Wei Ye[1], Wei Yu[2,*], Duo Li[1,*]

[+]Theses authors contributed equally to this study.

[1]Department of Food Science and Nutrition, Zhejiang University, Hangzhou, China

[2]The Province Center for Cardio-Cerebral-Vascular Disease, Zhejiang Hospital, Hangzhou, China

Table S1. Demographic and clinical characteristics in study population.

Characteristics	Case/n	Prevalence (%)	Crude OR (95% CI)	*P* value
Age (quartile), y				
35-48	200/592	33.78	1.00 (ref.)	
48-56	196/593	33.05	0.96 (0.76-1.23)	0.76
56-64	185/580	32.46	0.94 (0.74-1.20)	0.63
64-79	158/648	24.01	0.62 (0.48-0.80)	<0.001
Sex—n (%)				
Male—1153 (47.12)	360/1153	31.22	1.00 (ref.)	
Female—1294 (52.88)	388/1294	29.98	0.94 (0.79-1.12)	0.507
BMI (kg/m^2) —n (%)				
Normal—806 (32.94)	175/806	21.71	1.00 (ref.)	
Overweight—802 (32.77)	236/802	29.43	1.50 (1.20-1.88)	<0.001
Obesity—839 (34.29)	337/839	40.17	2.42 (1.94-3.00)	<0.001
Lifestyle factors —n (%)				
Smoking				
No (never/former)—1846 (75.81)	545/1846	29.52	1.00 (ref.)	
Yes (occasional/current)—601 (24.19)	203/601	33.78	1.26 (1.04-1.52)	0.015
Drinking				
No (never/former)——1781 (72.78)	498/1781	28.12	1.00 (ref.)	
Yes (occasional/current)—666 (27.22)	244/666	36.71	1.34 (1.12-1.60)	0.001
Education				
Primary—1630 (66.88)	558/1630	34.23	1.00 (ref.)	
Secondary—679 (27.86)	158/679	23.27	0.56 (0.35-0.92)	0.024
High—136 (5.25)	22/136	16.17	0.58 (0.47-0.73)	<0.001
Profession				
Mental labor—336 (14.20)	118/336	35.12	1.00 (ref.)	
Mixed labor—1116 (47.15)	364/1116	32.62 |	0.89 (0.69-1.15)	0.393
Manual labor—915 (38.66)	251/915	27.43	0.70 (0.53-0.91)	0.008
Exercise habit (times/week)				
No (< 3)—1959 (84.05)	642/2055	31.23	1.00 (ref.)	
Yes (≥ 3)—488 (15.95)	106/392	27.04	0.62 (0.50-0.78)	<0.001
Salt intake				
Low—1953 (80.50)	596/1953	30.52	1.00 (ref.)	
High—494 (19.50)	152/494	30.76	1.13 (0.87-1.45)	0.34
Animal oil intake				
No—1546 (63.59)	469/1546	30.34	1.00 (ref.)	
Yes—891 (36.41)	279/891	31.31	1.06 (0.89-1.26)	0.53
Clinical factors—n (%)				
Family history of hypertension				
No—1850 (75.61)	497/1847	26.92	1.00 (ref.)	
Yes—597 (24.39)	251/597	40.37	1.84 (1.51-2.23)	<0.001
Heart rate (beat/min)				

Low (<67)—807 (32.98)	234/807	29.00	1.00 (ref.)	
Moderate (67-74)—783 (32.41)	223/783	28.12	0.96 (0.77-1.19)	0.698
High (≥ 74)—847 (34.61)	291/847	34.36	1.28 (1.04-1.58)	0.019
TG (mmol/L)				
Normal (≤1.70) —1713 (70.00)	479/1713	28.81	1.00 (ref.)	
High (>1.70) —734 (30.00)	268/734	36.50	1.67 (1.32-2.12)	<0.001
TC (mmol/L)				
Normal (<5.18) —1779 (72.70)	515/1779	28.90	1.00 (ref.)	
High (≥5.18) —668 (27.30)	232/668	34.73	1.30 (1.07-1.59)	0.008
Fbg (mmol/L)				
Normal (<6.10) —2300 (94.30)	690/2300	29.67	1.00 (ref.)	
IFG (6.10-7.0) —60 (2.47)	18/60	32.73	1.91 (1.11-3.28)	
DM (≥ 7.0) —79(3.23)	29/79	37.50	1.94 (1.21-3.12	
Yes—126 (5.34)				

Table S2. Serum fatty acid and estimated desaturase index in participants with and without hypertension by gender status

FA and desaturase index*	Men (n=1153)				Women (n=1294)				*P* value[†]	*P* value[§]
	Hypertensive (n=360)	Normotensive (n=793)	Both (n=1153)	*P* value[#]	Hypertensive (n=388)	Normotensive (n=906)	Both (n=1294)	*P* value[#]		
16:00	21.07±2.59	20.81±2.55	20.89±2.57	0.10	21.04±2.55	20.46±2.63	20.63±2.62	0.01	0.18	0.18
18:00	6.36+0.79	6.48+0.92	6.45±0.78	0.25	6.40+0.80	6.33+0.92	6.38+0.88	0.02	0.05	0.01
16:1n-7	1.68+0.83	1.55+0.77	1.59+0.79	0.01	1.74+0.87	1.51+0.85	1.58±0.86	<0.01	0.80	0.42
18:1n-9	20.07±3.78	19.26±3.81	19.51±3.82	<0.01	19.93±3.79	18.75±3.79	19.10±3.83	<0.01	0.01	<0.01
18:3n-3	0.90±0.37	0.91±0.36	0.90±0.36	0.48	0.93±0.37	0.99±0.38	0.94±0.37	<0.01	<0.01	0.04
20:5n-3	3.24±1.57	3.34±1.62	3.27±1.61	0.36	3.21±1.63	3.23±1.67	3.22±1.64	0.84	0.44	0.36
22:5n-3	0.46 (0.38-0.56)	0.46 (0.38-0.56)	0.46 (0.37-0.56)	0.86	0.46 (0.38-0.56)	0.46 (0.38-0.56)	0.47 (0.38-0.58)	0.25	0.16	0.12
22:6n-3	1.66±0.60	1.74±0.61	1.71±0.61	0.04	1.67±0.60	1.76±0.62	1.73±0.62	0.01	0.43	0.26
18:2n-6	27.37±4.98	28.25±4.95	27.97±4.98	<0.01	27.60±5.49	28.92±5.57	28.52±5.28	<0.01	0.01	0.02
18:3n-6	0.33 (0.22-0.45)	0.31 (0.22-0.46)	0.32 (0.21-0.43)	0.42	0.38 (0.26-0.56)	0.36 (0.25-0.51)	0.35 (0.23-0.52)	<0.01	0.01	<0.01
20:3n-6	1.13±0.31	1.11±0.32	1.13±0.31	0.45	1.19±0.33	1.14±0.32	1.16±0.32	0.02	<0.01	0.03
20:4n-6	5.86±1.64	6.14±1.56	6.05±1.62	0.01	5.92±1.63	6.08±1.53	6.00±1.60	0.10	0.43	0.35
SCD-1 ($\times 10^{-2}$)	7.31 (5.32-9.59)	6.65 (5.10-8.97)	6.81 (5.17-9.14)	0.02	7.44 (5.55-10.23)	6.62 (4.92-9.01)	6.59 (4.71-9.08)	<0.01	0.68	0.47
SCD-2	3.20±0.74	3.06±0.76	3.12±0.76	<0.01	3.16±0.76	2.94±0.74	3.00±0.75	<0.01	<0.01	0.02
D5D	5.18±1.95	5.54±1.87	5.44±1.92	<0.01	5.44±1.84	5.73±1.96	5.64±1.93	0.02	0.01	<0.01
D6D ($\times 10^{-2}$)	1.25 (0.76-1.82)	1.10 (0.74-1.68)	1.13 (0.75-1.71)	0.08	1.44 (0.90-2.24)	1.19 (0.71-1.88)	1.27 (0.77-1.98)	<0.01	<0.01	0.01

FA, fatty acid; SCD-1, 16:1n-7/16:0; SCD-2, 18:1n-9/18:0; D5D, 20:4n-6/20:3n-6; D6D, 18:3n-6/18:2n-6.

*Data with normal distribution were expressed as the mean (s.d.), while the skewed data were expressed as the median (quartile rage) and were log-transformed before data analyses.

[#]*P* value b between genders was calculated by unpaired t-test

[†] *P* value between genders was calculated by a non-adjusted GLM.

[§] *P* value between genders was calculated by age, BMI and hypertension-adjusted GLM.

Table S3. Varimax rotation loadings of the 3 major principal fatty acid–component

Fatty acid (FA)	Comp 1 (D6D component)	Comp 2 (SCD-2 component)	Comp 3 (D5D component)
16:0	**0.44**[a]	-0.07	-0.44
18:0	0.17	**-0.55**[b]	-0.17
16:1n-7	**0.65**[a]	0.26	-0.28
18:1n-9	0.17	**0.86**[b]	-0.25
18:2n-6	**-0.67**[a]	-0.43	-0.09
18:3n-6	**0.84**[a]	-0.06	0.02
20:3n-6	**0.53**[a]	-0.23	-0.33[c]
20:4n-6	0.10	-0.34	**0.73**[c]
18:3n-3	-0.04	**0.40**[b]	-0.21
20:5n-3	0.13	0.22	**0.55**[c]
22:5n-3	**0.56**[a]	-0.04	0.26
22:6n-3	0.10	-0.36	**0.39**[c]
SCD-1	**0.47**	0.08	-0.14
SCD-2	0.10	**0.93**[b]	-0.12
D5D	-0.25	-0.11	**0.89**[c]
D6D	**0.90**[a]	0.07	0.03
Eigenvalue	3.42	2.78	1.46
Proportion of total variance (%)	37.80	17.86	12.98

*Values in boldface were major factor loadings that contribute to identify Comp 1.
[#]Values in boldface were major factor loadings that contribute to identify Comp 2.
[†]Values in boldface were major factor loadings that contribute to identify Comp 3.

Table S4. Multivariate-adjusted odds ratios (ORs) for prevalent hypertension in the highest compared with the lowest quartile of principal component scores stratified by age, gender and BMI

Factors stratified	case/n	Comp 1		Comp 2		Comp 3	
		OR (95% CI)	*P* for interaction*	OR (95% CI)	*P* for interaction*	OR (95% CI)	*P* for interaction*
Gender			0.15		0.88		0.59
Women	388/1294	1.71 (1.13-2.57)		1.54 (0.85-2.78)		0.53 (0.35-0.80)	
Men	360/1153	1.21 (0.79-1.85)		1.20 (0.66-2.18)		0.78 (0.52-1.18)	
Age			0.11		0.59		0.68
≤55	396/1205	1.20 (0.80-1.79)		1.36 (1.17-1.58)		0.66 (0.45-1.00)	
>55	352/1242	1.91 (1.24-2.91)		0.96 (0.82-1.25)		0.62 (0.41-0.92)	
BMI			0.78		0.73		0.52
≤24	317/1332	1.65 (1.09-2.49)		1.37 (0.72-2.60)		0.68 (0.46-1.01)	
>24	431/1115	1.68 (1.02-2.77)		1.51 (0.85-2.68)		0.58 (0.40-0.87)	

**P* for interaction was estimated to determine whether prevalent OR differed between the strata analyzed by including simultaneously each strata factor, the quartiles of principal component scores and the respective interaction terms (strata factor multiplied by quartiles of principal component scores) in multivariable logistic regression models.
